# MPP7 as a Novel Biomarker of Esophageal Cancer: MPP7 Knockdown Inhibits Esophageal Cancer Cell Migration and Invasion

**DOI:** 10.3390/life12091381

**Published:** 2022-09-05

**Authors:** Zhaodong Li, Yongyao Tang, Jing Cai, Shunlong Wu, Fangzhou Song

**Affiliations:** 1 Molecular Medicine and Cancer Research Center, Chongqing Medical University, 1#Yixue Yuan Road, Yuzhong District, Chongqing 400016, China; 2 Department of Oncology, The First Affiliated Hospital of Chongqing Medical University, Chongqing 400016, China

**Keywords:** MPP7, esophageal cancer, biomarker, migration, invasion

## Abstract

MAGUK p55 scaffold protein 7 (MPP7) is a member of the stardust family of membrane-associated guanosine kinase protein P55 and plays a role in the establishment of epithelial cell polarity. However, its potential implication in human esophageal cancer is unclear. In this study, we investigated the expression profile of MPP7 and its functional impact on esophagus cancer. Expression analyses of immunohistochemical microarrays with survival and prognostic information of 103 patients with esophageal cancer demonstrated that MPP7 was overexpressed in 52 patients, who showed poor survival rates. The transcriptional expression of MPP7 in esophageal cancer in TCGA database increased successively from normal epithelial, to esophageal adenocarcinoma, to esophageal squamous cell carcinoma. Transcriptome sequencing after MPP7 knockdown in esophageal carcinoma cells showed that wound-healing-associated proteins were down-regulated, and the TGF-β pathway was one of the important signaling pathways. A loss-of-function study showed that the knockdown of MPP7 inhibited cell migration and invasion. These results could be verified in a model of tumor cells injected into the tail vein and subcutaneous tumor of nude mice. Herein, our results indicated that MPP7 could have an oncogenic role in human esophagus cancer, thus demonstrating its potential as a novel biomarker for the diagnosis and/or treatment of esophagus cancer.

## 1. Introduction

Esophageal cancer is one of the deadliest cancers in the world, mainly because of its extreme aggressiveness and low survival rates. It is the sixth leading cause of cancer death and the eighth most common cancer in the world [1]. However, unlike western countries, the majority of esophageal cancer pathologies in China and Japan are squamous cell carcinoma. The possible causes of the disease are eating habits involving pickled food and too-hot and overheated food [2]. The diagnosis of esophageal cancer mainly relies on gastroscopy, and there is still a lack of specific biomarkers. Therefore, we analyzed and identified MAGUK p55 scaffold protein 7 (MPP7), which is highly expressed in esophageal cancer patients, leading to poor survival rates, using immunohistochemical microarray and bioinformatics.

MPP7 is a member of the stardust family of membrane-associated guanosine kinase protein P55 and plays a role in the establishment of epithelial cell polarity [3]. Tucke, V.M., confirmed that MPP7 is a PDZ protein that interacts with human papillomavirus 16-E6 and forms a tripartite complex with LIN7A or LIN7C and DLG1, regulating the stability and localization of DLG1 in cell connections [4]. MPP7 is a novel candidate gene for primary open-angle glaucoma (POAG), and there is evidence that its expression in relevant eye tissues and its dysregulation under mechanical stress may mimic disease scenarios [5]. The ectopic expression of MPP7 significantly enhanced the migration and invasion ability of breast cancer cells. MPP7 exerts its promotive effect by regulating epithelial mesenchymal transformation (EMT) and activating the EGFR/AKT cascade [6]. However, to date, the expression of MPP7 and its potential functional impact on esophageal cancer have not been investigated and remain elusive. Based on the close relationship between esophageal squamous epithelial cells and cell polarity [4], we speculated that MPP7 plays an important role in the pathogenesis of esophageal cancer, so we conducted the studies here described.

In this study, we investigated the expression of MPP7 and its functional impact on esophageal cancer for the first time. Expression analyses of immunohistochemical microarrays with survival and prognostic information of 103 patients with esophageal cancer demonstrated that MPP7 was overexpressed in 52 patients, who showed poor survival rates. MPP7 knockdown in esophageal carcinoma cells with transcriptome sequencing showed that wound-healing-associated proteins were down-regulated, and the TGF-beta pathway was one of the important signaling pathways. The transcriptional expression of MPP7 in esophageal cancer in TCGA database increased successively from normal epithelial cells, to esophageal adenocarcinoma, to esophageal squamous cell carcinoma. A loss-of-function study showed that MPP7 inhibited cell migration and invasion. Western blot assays verified that the signaling pathway of MPP7 affecting esophageal cancer cells could be the TGF-beta pathway, which was consistent with the results of transcriptomic sequencing. In vivo, MPP7 knockdown reduced cell migration via the injection of tumor cells into the tail vein of nude mice. By subcutaneous tumor loading in nude mice, we found that the basal invasion of the experimental group was reduced. In summary, our results indicated that MPP7 could have an oncogenic role in human esophagus cancer, thus demonstrating its potential as a novel biomarker for the diagnosis and/or treatment of esophagus cancer.

## 2. Materials and Methods

### 2.1. Cell Culture and Cell Lines

Human esophagus cancer line KYSE-150 was purchased from Cell Bank of Chinese Academy of Sciences. The KYSE-150 cell line was cultured in Dulbecco’s modified Eagle’s medium (DMEM; Gibco, Waltham, MA, USA) supplemented with 10% fetal bovine serum (FBS; Gibco, Waltham, MA, USA), at 37 °C, in a 5% CO_2_ incubator (Thermo, Waltham, MA, USA).

### 2.2. RNA Interference (RNAi)

KYSE-150 cells were transfected with 80 pmol MPP7 siRNA (GenePharma Shanghai, China) using Lipofectamine 2000 (Invitrogen; Thermo Fisher Scientific, Waltham, MA, USA). The sequences were: control siRNA duplex,

Sense: 5-UUCUCCGAAUGUGUCACATTT-3;

Antisense: 5-ACGGUACACGUTCGGAGAATT-3;

MPP7 siRNA(472),

Sense: 5-GCCUGCAUUCAUUGGUAAAGATT-3;

Antisense: 5-GCCUGCAUUCAUUGGUAAAGATT-3.

### 2.3. Total RNA Extraction and Real-Time Polymerase Chain Reaction (qRT-PCR)

Total RNA from esophagus cancer cells was extracted using TRIzol^®^ reagent (Takara Bio, Dalian, China) according to the manufacturer’s instructions. RNA quality and concentration were determined by determining the absorbance with a NanoDrop 2000 spectrophotometer (Thermo Fisher Scientific, Waltham, MA, USA). Reverse transcription (RT) of first-strand cDNAs (1 µg) was performed using PrimeScript RT Master Mix (Perfect Real Time; Takara Bio, Dalian, China) according to the manufacturer’s protocol. All quantitative data were normalized to GAPDH using the 2^−ΔΔCq^ method. The primer sequences were: forward primer, CCTTCCTCTGGGATATGTTTGGT; reverse primer, AGCCTTCACATTGGGTTTTGA.

### 2.4. Western Blot Analysis

Total proteins were extracted from cultured cells. Cell lines were permitted to grow until they reached 95% confluence. After two washes with PBS, cells were lysed with a general lysis buffer (NP40; Beyotime, Nantong, China) to generate the total-protein lysate. Proteins were then subjected to 10% sodium dodecyl sulfate polyacrylamide gel electrophoresis (SDS-PAGE) with loading of equal amounts of whole proteins (50 μg) per lane. β-Actin was simultaneously detected as a loading control. Immunoreactivity was determined with enhanced chemiluminescence autoradiography using the following antibodies: FGFR2 antibody, MPP7 antibody, and TGFB2 antibody, which were all purchased from Proteintech (Proteintech, Wuhan, China) (1:2000), and horseradish-peroxidase-conjugated secondary antibodies (1:4000).

### 2.5. Wound-Healing Assay

KYSE-150 cells were seeded into six-well plates (5 × 10^5^ cells/well) and transfected with siMMP7 or control siRNA. A sterile 20 μL pipette tip was used to scrape across the center of each well 48 h post transfection. Subsequently, cells were rinsed with phosphate-buffered saline (PBS) for three times and immediately cultured in full-serum medium. The distance between the scratches was observed and measured at 24 h, 48 h, and 72 h, respectively.

### 2.6. Transwell Assays

KYSE-150 cells were cultured in 24-well plates and transfected with specific siMPP7 or control siRNA. Forty-eight hours post transfection, cells were harvested, and single-cell suspensions in serum-free DMEM were prepared, of which 150 μL (3 × 10^4^ cells) was seeded into the upper chambers of an 8 µm Transwell plate (Corning Life Sciences, Corning, NY, USA). The lower chamber was filled with 600 μL of DMEM supplemented with 10% FBS. For the invasion assay, the membrane was coated with Matrigel (Beyotime, Nantong, China) 6 h prior to seeding. Following incubation at 37 °C for 24 h, cells were fixed with ice-cold methanol for 20 min and stained with 0.1% crystal violet for 5 min at room temperature.

## 3. Immunohistochemical and Clinical Data Analysis

An immunohistochemical chip was purchased from Shanghai Super Chip Company (Shanghai, China) and contained the detailed clinical prognostic data of 103 patients with esophageal cancer. The data included 85 men and 15 women, all with squamous cell carcinoma. The minimum age was 49, and the maximum age was 85. Paraffin sections were successively dewaxed, antigen-repaired, serum-sealed, and incubated with a primary antibody at 4 °C overnight (1:200; Proteintech); then, they were incubated with a secondary antibody at 37 °C for half an hour and cleaned with PBS for three times; chromogenic agent was added, and the samples were sealed. According to the product of dyeing intensity and dyeing area, the full score was 12 points. A Cox regression analysis was performed to analyze the results of the immunohistochemistry score combined with clinical data, and the survival prognosis of MPP7 patients was analyzed. TCGA esophageal transcriptome data and clinical data were obtained from the GDC database (https://portal.gdc.cancer.gov/) (accessed on 2 March 2022). The expression level of the FPKM gene was used to analyze the differential expression of genes in squamous cell esophageal carcinoma, esophageal adenocarcinoma, and normal esophageal epithelium. In order to reduce the false positives, values outside the 10~90th percentile interval of the group were considered outliers and excluded from this analysis when we analyzed the gene differential expression among each group.

### 3.1. HE Pathological Section

Pathological specimens were obtained from subcutaneous-tumor-bearing nude mice. The main steps for obtaining HE-stained pathological sections were as follows: (1) The specimen was placed in 10% formalin for 24 h. (2) The specimen was washed in water for 10 min. (3) The specimen was dehydrated with 75% alcohol for 1.5 h at 35 °C. (4) The specimens were made transparent with xylene twice, 1 h each time. (5) The samples were immersed in paraffin for three times, 1 h each time, at 55 °C. (6) The samples were embedded and sliced. The slices were 4 microns thick. (7) The slices were baked at 65 °C for 30 min and then placed in xylene for dewaxing for three times. (8) The slices were stained with sappanwood purple for 5 min and eosin for 20 s. (9) Sealing piece.

### 3.2. Transcriptome Sequencing and Analysis

KYSE-150 cells were cultured in 6-well plates and transfected with specific siMPP7 or control siRNA. There were three samples in each group, and GenePharma company (GenePharma, Shanghai, China) was commissioned to conduct transcriptome sequencing and analyze relevant data. Key steps included: (1) Sequencing data statistics: The transcriptome analysis of nine samples was completed, and 104.182 Gb of clean data (sequencing data after quality control) was obtained. The average amount of clean data of each sample was 11.576 Gb. The percentage of Q30 base was above 92.80%, and GC content was between 48.50% and 49.82%. (2) Comparison with reference genome: Reference species, human; reference genome version, GRCh38; reference genome source, http://asia.ensembl.org/homo_sapiens/Info/Index. The sequencing data of each sample after quality control were compared with the specified reference genome, and the comparison rates ranged from 95.274% to 95.593%, among which the unique comparison rates ranged from 83.737% to 91.903%. (3) Expression analysis: Known genes’ annotated source, http://asia.ensembl.org/homo_sapiens/Info/Index; annotated version of known genes, release-94; known genes’ annotated source, http://asia.ensembl.org/homo_sapiens/Info/Index. A total of 58,735 genes were involved in expression analysis, including 58,735 known genes (32,961 expressed) and 0 new genes. There were 206,601 transcripts, including 206,601 known transcripts (126,155 expressed transcripts) and 0 new transcripts. (4) Analysis of expression differences: Based on the quantitative expression results of genes or transcripts, the differences between groups were analyzed to obtain genes or transcripts with differential expression between the two groups. Bioinformatics was used to analyze the above data through the GEO database or TCGA data to predict the possible signaling pathways of MPP7.

### 3.3. Tumor Cells Were Injected into Subcutaneous-Tumor-Bearing Model and Caudal Vein in Nude Mice

Athymic BALB/c mice (14 females) aged six weeks were purchased from KA WEN SI BAI GE Laboratory Animal Company (Suzhou, China) and were housed in laminar airflow chambers under specific pathogen-free conditions. KYSE-150 cells transfected with LV-shMPP7 or LV-shNC (GenePharma, Shanghai, China) were injected into the caudal vein of eight mice (1 × 10^7^ cells/100 microliter); each group had four mice. KYSE-150 cells transfected with LV-shMPP7 or LV-shNC (GenePharma, Shanghai, China) were subcutaneously injected into six mice (1 × 10^7^ cells/100 microliter); each group had three mice. Four weeks post injection, all mice were sacrificed. Fluorescence imaging was performed on nude mice that were injected into the caudal vein to determine the intensity of the fluorescence signal. The intensity and area of the fluorescence signal represented the range and density of tumor metastasis; Image J was used for grading. Average fluorescence intensity (Mean) = Total fluorescence intensity (IntDen)/Area (Area). Subcutaneous-tumorigenesis specimens were obtained, and HE pathological sections were stained to observe the degree of tumor invasion to the base. All nude mice were treated humanely throughout the experiment and were killed painlessly.

### 3.4. Statistical Analysis

Data were expressed as means ± standard deviation (SD). All statistical analyses were performed using SPSS 17.0 software (SPSS, Chicago, IL, USA). The comparisons between groups were performed with Student’s *t*-test, Fisher’s exact tests, or the χ^2^ test. Survival curves were plotted in accordance with the Kaplan–Meier method and were analyzed with the log-rank test. *p* < 0.05 was considered statistically significant.

## 4. Results

### 4.1. Clinicopathological and Prognostic Significance of MPP7 Expression in Esophagus Cancer

**Figure 1:**
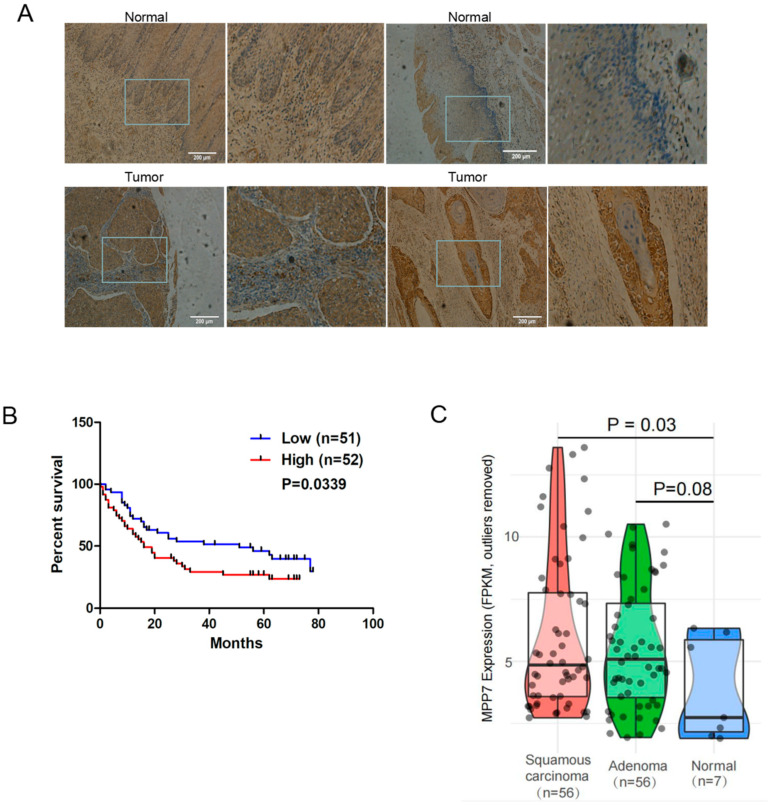
(**A**) Immunohistochemical microarrays containing the survival and prognosis information of 103 patients with esophageal cancer. Combined with staining scores, the expression level in cancer was higher than that in normal tissues (*p =* 0.02). (**B**) Kaplan–Meier survival analysis showed that the overall survival rates over 5 years of the high-MPP7 group were lower than those of the low-MPP7 group (*p* = 0.0339). (**C**) Data extracted from TCGA showed that the expression level of MPP7 in 56 patients with esophageal squamous cell carcinoma was significantly higher than that in normal controls (*p* = 0.03).

Immunohistochemistry showed that MPP7 was overexpressed in esophagus cancer tissues and was negative in normal tissue (Figure 1A and Table 1) (*p* = 0.02). As shown in Figure 1B, the Kaplan–Meier survival analysis showed that the overall survival rates over 5 years of the high-MPP7 group (*n* = 52) were lower than those of the low-MPP7 group (*n* = 51) (*p* = 0.0339). TCGA database showed that the transcriptional expression level of MPP7 increased successively from normal epithelium, to esophageal adenocarcinoma epithelium, to esophageal squamous cell carcinoma (*p* = 0.03) (Figure 1C). Based on the clinical follow-up data of 103 patients, the multivariate Cox regression analysis suggested that MPP7 was associated with age (≥60 vs. <60; *p* = 0.007), N stage (N0 vs. N1–3; *p* = 0.002), and clinical stage (I–II vs. III–IV; *p* = 0.002) (Table 1). These data suggested that MPP7 was up-regulated in human esophagus cancer and may be a poor prognostic indicator.

### 4.2. MPP7 Knockdown in Esophagus Cancer Cell Inhibited Migration and Invasion

**Figure 2:**
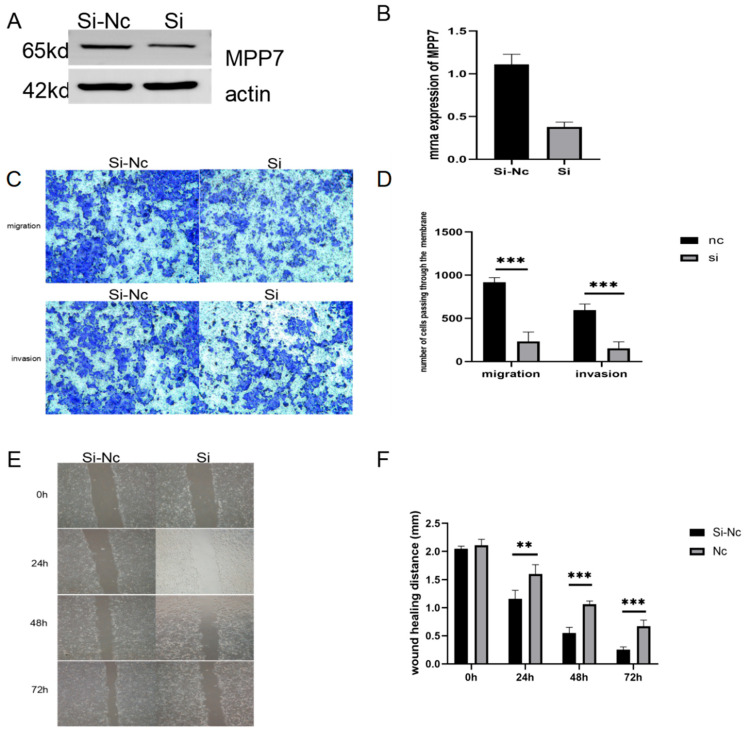
(**A**,**B**) SiRNA effectively interfered with esophageal carcinoma KYSE-150 at both mRNA and protein levels. (**C**,**D**) Transwell assay confirmed that MPP7 knockdown inhibited the migration and invasion of cells. In the migration experiment, the number of cells crossing the membrane was 972 ± 10.408 in the control group and 248 ± 9.29 in the experimental group (*p* < 0.05). In the invasion experiment, the number of cells crossing the membrane was 590 ± 9.5 in the control group and 235 ± 5.5 in the experimental group (*p* < 0.05). (**E**,**F**) Wound-healing experiment confirmed that the cell-migration ability in the experimental group significantly decreased after MPP7 was knocked down. At the observation end point of 72 h, the control group showed values of 0.253 ± 0.302 (mm), and the experimental group showed values of 0.67 ± 0.11 (mm) (*p* < 0.05). (**: *p* < 0.01, ***: *p* < 0.001).

Most malignant tumor cells have strong migration and invasion ability, which is a necessary condition for the distant metastasis of tumor. Quantitative RT-PCR and Western blot analysis demonstrated that MPP7 expression was significantly inhibited at both the mRNA and protein levels 48 h after transfection via siRNA-mediated silencing in KYSE-150 cells (Figure 2A,B) (*p* < 0.05). We confirmed that MPP7 knockdown in esophageal cancer cell line KYSE-150 significantly reduced cell migration and invasion (Figure 2C,D). At the same time, the migration ability of esophageal cancer cells was significantly decreased after MPP7 was inhibited in wound-healing experiment (Figure 2E,F) (*p* < 0.05). (***: *p* < 0.001).

### 4.3. Investigation of the Effect of MPP7 on the Invasion and Migration of Esophageal Cancer Cells in Nude Mice

**Figure 3:**
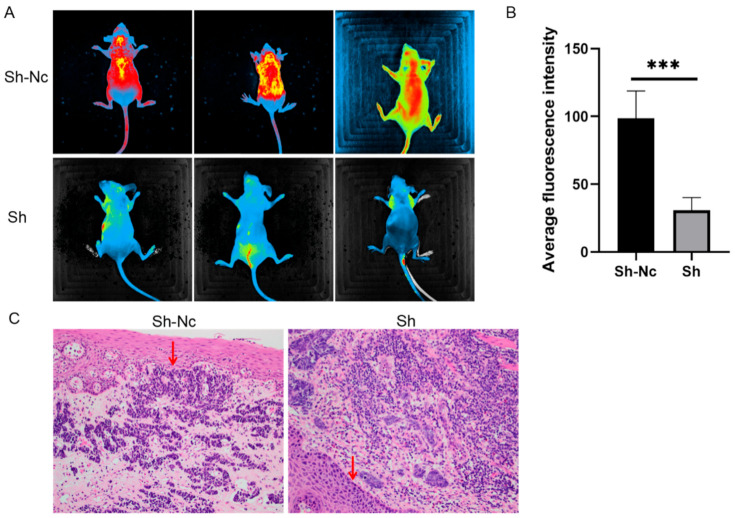
(**A**,**B**) Esophageal carcinoma cells with stable and low expression of MPP7 were injected into the tail vein of nude mice, and their distant metastasis ability and intensity were significantly lower than those of the control group *(p* < 0.001). (**C**) In the subcutaneous-tumor-bearing model of nude mice, epidermal invasion by implant tumor in the experimental group was significantly lower than that in the control group (area indicated by red arrow). (***: *p* < 0.001).

We used a subcutaneous-tumor-bearing model and a tail-vein-injection model of nude mice to verify the invasion and migration of tumor cells. The area and intensity of fluorescence in the rest of the body of nude mice with stable and the low expression of MPP7 were significantly smaller than those in the control group. This means that esophageal cancer cells with MPP7 knockdown had a decreased ability to metastasize in nude mice (*p* = 0.0006) (Figure 3A,B). In the subcutaneous-tumor-bearing model of nude mice, the invasion of the basal membrane in the experimental group was significantly lower than that in the control group (Figure 3C).

### 4.4. Transcriptome Sequencing and Signaling Pathway Validation

**Figure 4:**
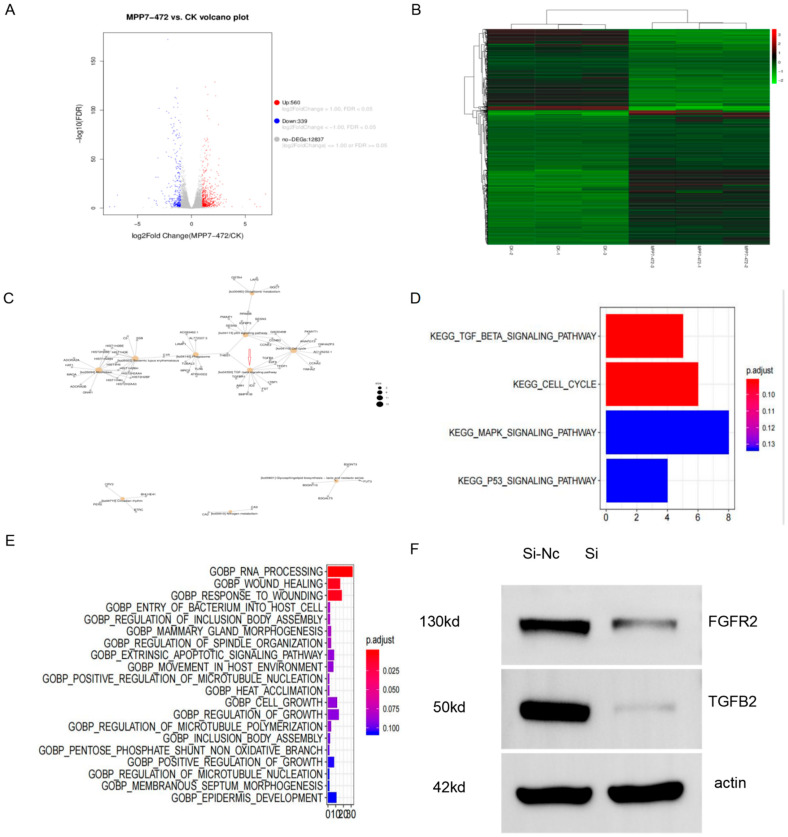
(**A**,**B**) A total of 560 differentially expressed genes were significantly up-regulated, and 339 genes were down-regulated. (**C**,**D**) The TGF-β signaling pathway was down-regulated after MPP7 was inhibited. The TGF-β signaling pathway was significantly correlated with MPP7. (**E**,**F**) The enrichment analysis of GO biological processes suggested that wound healing was one of the down-regulated functional plates, and the expression levels of FGFR2 and TGF-β decreased, which was consistent with bioinformatic predictions.

We used siRNA MPP7-472 to inhibit the expression of MPP7 in esophageal cancer cells KYSE-150, followed by transcriptome sequencing. We performed bioinformatic analyses of differential gene expression and signaling pathways. Subsequently, a Western blot was used to verify relevant data. As shown in Figure 4A,B, a total of 560 differentially expressed genes were significantly up-regulated, and 339 genes were down-regulated. The enrichment analysis of GO biological processes suggested that wound healing was one of the down-regulated functional plates (Figure 4E). Subsequently, a Western blot was used to verify the TGF-beta pathway. The relative expression levels of TGF-β and FGFR decreased, which was consistent with the transcriptome sequencing results (Figure 4F). KEGG signaling-pathway enrichment indicated that the TGF-β pathway was one of the important, related signaling pathways, and the TGF-β signaling pathway was down-regulated after MPP7 was inhibited (Figure 4C,D).

## 5. Discussion

The early diagnosis rate of esophageal cancer is not high; diagnosis requires careful screening via magnifying endoscopy and staining endoscopy and lacks specific serological diagnostic indicators. At the same time, middle-stage and advanced esophageal cancer is generally not sensitive to chemotherapy, so it is of great clinical significance to find new biological targets [7]. So far, many biological targets have been reported for the diagnosis and treatment of esophageal cancer but have not been used in the clinical setting. Liu, X.S., reported that GLUT1, which is related to tumor immune infiltration, can be used as a biomarker for the diagnosis and treatment of esophagus cancer [8]. LncRNA AK058003 is highly expressed in esophageal cancer patients and promotes the proliferation, migration, invasion, and metastasis of esophageal cancer cells [9]. Based on the fact that MPP7 is an important protein regulating epithelial cell polarity and that epithelial polarity is closely related to the occurrence and development of tumor [10], we studied the immunohistochemical microarrays of 103 patients with esophageal cancer to obtain survival and prognosis data, and the results suggested that MPP7 was highly expressed in tumor tissues. A Cox multivariate regression analysis indicated that MPP7 was associated with age, N stage, and clinical stage. TCGA data also showed that the transcription level of MPP7 increased gradually from normal epithelium, to esophageal adenocarcinoma, to esophageal squamous cell carcinoma. At the same time, the inhibition of MPP7 both in vivo and in vitro showed a decrease in cell invasion and migration. These results suggest that MPP7 may be a potential diagnostic and prognostic indicator of esophageal cancer. However, due to the limitations of clinical data, further serological tests are needed to provide more evidence to support MPP7 as a target for clinical applications. However, since most esophageal cancers in China are cell squamous cell carcinoma, we were not able to obtain the expression and clinical significance of MPP7 in esophageal adenocarcinoma, and relevant data are expected to be further supplemented in the future.

Cell polarity is defined as the asymmetry of cell shape, organelle distribution, and cell function. Cell polarity is critical for cell migration and invasion [11,12]. In addition, cell polarity is also required during migration, development, and wound healing. The correct regulation of polarity signals is also important for maintaining the apical–basal polarity of epithelial cells, the loss of which is a prerequisite and marker for cancer [13,14]. In our study, including in vivo and in vitro analyses, MPP7 knockdown could reduce the migration and invasion abilities of esophageal cancer. MPP7 plays an important role in regulating cell polarity [3], and esophageal cancer has the abilities to easily invade basement membrane and to perform distant lymph-node metastasis [15]. Therefore, MPP7 has the potential to be a target gene in the treatment of esophageal cancer.

Transcriptome sequencing further clarified the specific mechanism and signaling pathway of MPP7 in esophageal cancer. The results of cell functional enrichment were mainly concentrated in wound healing, which was consistent with our experimental results. The bioinformatic enrichment analysis after transcriptome sequencing was consistent with the basic results, which proved that our experimental results were real and credible. In the meantime, in order to verify the credibility of bioinformatics, a Western blot was used to verify the TGF-β pathway, and the TGF-β pathway was down-regulated, which was consistent with the transcriptional sequencing results. Transforming growth factor β(TGF-β) is a multifunctional peptide cytokide that can regulate cell proliferation, differentiation, migration, apoptosis, adhesion, angiogenesis, immune surveillance, and survival. TGF-β overexpression in malignant tumors promotes tumor cell invasion and metastasis by promoting angiogenesis and inflammation, inducing immune escape, promoting epithelial–mesenchymal transformation, and inducing other mechanisms [16,17]. TGF-β can form a synergy with the Ras pathway, which is a key inducer of EMT, and can promote tumor cells to promote invasion and metastasis. Studies have shown that the disruption of cell polarity may work in conjunction with TGF-β signaling or promote TGF-β-mediated EMT [18]. The Crumbs-mediated disruption of the complex predisposes Eph4 cells to EMT, which are normally insensitive to TGF-β mediated EMT [19]. After the knockdown of MPP7, TGF-β decreases, which may further affect EMT through some of the above mechanisms. At the same time, the effect of MPP7 on cell polarity also affects TGF-β. This dual mechanism may jointly participate in the regulation of EMT, but the specific in-depth mechanism still needs to be further studied, which is to be our research direction and focus.

In conclusion, MPP7 was highly expressed in esophageal carcinoma and could regulate the migration and invasion of esophageal squamous cell carcinoma by affecting cell polarity and the TGF-β signaling pathway. MPP7 is expected to become a new therapeutic target and prognostic marker of esophageal squamous cell carcinoma.

## Figures and Tables

**Table 1:** Based on the survival and prognosis information of the patients contained in the immunohistochemical chip, combined with the staining scores, the Cox regression analysis suggested that MPP7 was associated with age (≥60 vs. <60; *p* = 0.007), N stage (N0 vs. N1–3; *p* = 0.002), and clinical stage (I–II vs. III–IV; *p* = 0.002).

Clinical Variable	Comparison	HR	95% CI I	*p*
**Age**	≥60 vs. <60	**2.547**	**1.285–5.047**	**0.007**
**Gender**	Male vs. Female	2.117	0.879–5.099	0.095
**Histological grade**	I/II vs. III	1.276	0.660–2.467	0.468
**T stage**	T1–2 vs. T3–4	1.521	0.725–3.188	0.267
**N stage**	N0 vs. N1–3	**2.446**	**1.391–4.299**	**0.002**
**Tumor size**	≥5 vs. <5	1.305	0.750–2.273	0.346
**Clinical stage**	I–II vs. III–IV	**2.446**	**1.391–4.299**	**0.002**
**X in cancer**	Low vs. High	**1.888**	**1.105–3.224**	**0.020**

## Data Availability

Not applicable.

## Abbreviations

TCGA	The Cancer Genome Atlas
KEGG	Kyoto Encyclopedia of Genes and Genomes
MPP7	MAGUK p55 scaffold protein 7
DLG1	discs large MAGUK scaffold protein 1
POAG	Primary open-angle glaucoma
EMT	Epithelial mesenchymal transformation
GLUT1	Glucose transporter 1
LIN7C	lin-7 homolog C, crumbs cell polarity complex component
LIN7A	lin-7 homolog A, crumbs cell polarity complex component
EGFR	Epidermal Growth Factor Receptor
Mean	Mean gray value
IntDen	Integrated density
